# An in-vitro evaluation of the flow haemodynamic performance of Gore-Tex extracardiac conduits for univentricular circulation

**DOI:** 10.1186/s13019-020-01269-x

**Published:** 2020-09-02

**Authors:** Shane McHugo, Lars Nolke, Patrick Delassus, Eugene MacCarthy, Liam Morris, Colin Joseph McMahon

**Affiliations:** 1 Galway Medical Technology Centre, Department of Mechanical and Industrial Engineering (GMIT), Galway, Ireland; 2 Department of Cardiothoracic Surgery, Children’s Health Ireland, Crumlin Dublin 12, Ireland; 3 Department of Pediatric Cardiology Children’s Health Ireland, Crumlin Dublin 12, Ireland; 4 University College Dublin School of Medicine, Belfield Dublin 4, Ireland

**Keywords:** Additive manufacturing, 3D printing, Fontan, Total cavopulmonary connection, Cardiac surgery

## Abstract

**Objective(s):**

The Fontan procedure is a common palliative intervention for sufferers of single ventricle congenital heart defects that results in an anastomosis of the venous return to the pulmonary arteries called the total cavopulmonary connection (TCPC). In patients with palliated single ventricular heart defects, the Fontan circulation passively directs systemic venous return to the pulmonary circulation in the absence of a functional sub-pulmonary ventricle. Therefore, the Fontan circulation is highly dependent on favourable flow and energetics, and minimal energy loss is of great importance. The majority of in vitro studies, to date, employ a rigid TCPC model. Recently, few studies have incorporated flexible TCPC models, without the inclusion of commercially available conduits used in these surgical scenarios.

**Method:**

The methodology set out in this study successfully utilizes patient-specific phantoms along with the corresponding flowrate waveforms to characterise the flow haemodynamic performance of extracardiac Gore-Tex conduits. This was achieved by comparing a rigid and flexible TCPC models against a flexible model with an integrated Gore-Tex conduit.

**Results:**

The flexible model with the integrated Gore-Tex graft exhibited greater levels of energy losses when compared to the rigid walled model. With this, the flow fields showed greater levels of turbulence in the complaint and Gore-Tex models compared to the rigid model under ultrasound analysis.

**Conclusion:**

This study shows that vessel compliance along with the incorporation of Gore-Tex extracardiac conduits have significant impact on the flow haemodynamics in a patient-specific surgical scenario.

## Introduction

The total cavopulmonary connection (TCPC) is a modification of the original surgical repair of tricuspid atresia introduced by Francis Fontan [1] and is used for the management of single ventricle congenital heart disease. This procedure, which involves the anastomosis of the inferior (IVC) and superior vena cava (SVC) directly to the unbranched pulmonary artery (excluding most of or all the right atrium) has been the focus of recent studies in an attempt to reduce postoperative complications and improve functional outcome for patients. Repair of these defects currently requires a series of complex procedures within the first 2–4 years of life collectively referred to as staged Fontan palliation [2]. The end goal of these procedures is to reroute systemic venous blood directly to the lungs without returning to the heart. The first stage is the Norwood procedure performed shortly after birth, followed a few months later by the Glenn or hemi-Fontan procedure. The final stage, the Fontan procedure, bypasses the right ventricle to create a direct connection between the vena cavae and the pulmonary arteries [3]. After Fontan completion, the right ventricle is committed to pump blood to the body and then the lungs in series, doubling the workload imposed on the single ventricle. The lungs are perfused passively by venous pressure alone, creating inefficiencies that lead to a characteristic constellation of complications including low cardiac output, progressive ventricular failure, protein-losing enteropathy, plastic bronchitis, and activity intolerance [4]. Combined, these effects lead to morbidities that reduce the quality and length of life.

Energy-consuming abnormal blood flow patterns such as flow separation, flow collision, or helical flow [5] caused by geometrical effects can increase the working load to the ventricles in maintaining adequate flow throughout the cardiovascular system, which may ultimately result in heart failure [6]. The degree of energy loss, and thereby its hemodynamic impact, is conventionally assessed with indirect, global parameters, such as vessel size, pressure gradients or effective orifice area, which may lead to inaccurate disease severity characterization [6–9].

Although in-vitro modelling plays an important role in the research of single ventricle energy loss, it should be realized that the early models, which demonstrated the importance of geometric factors such as caval offsetting and flaring on energy loss, included highly simplified, symmetrical, cross-like rigid tubes with uniform diameters [10–13]. Implementing more physiological features in these models, such as non-uniform vessel diameters of the PAs, can significantly change fluid hemodynamic parameters [14]. Besides the limiting, simplified geometry, most models applied a steady inflow conditions and rigid wall assumptions [15–17]. Patient-specific MRI-based TCPC models have led to more accurate models, although these models still apply rigid walls and steady inflow conditions [18–20]. The clinical relevance of energy loss HLHS to date has shown that increased energy loss is linked to (1) reduced exercise capacity, and (2) altered cardiac parameters and increased central venous pressure (CVP).

To date, there is still no perfect conduit for reconstruction of paediatric cardiothoracic surgical scenarios [21]. This investigation aims to experimentally identify the impact of an extracardiac Gore-tex conduit on single ventricle palliation with respect to haemodynamic parameters, within a patient-specific thin-walled flexible model. The assumption of applying a rigid wall solution for TCPC simulations will also be addressed.

## Materials and methods

One anonymised patient specific medical image dataset of the TCPC circulation, post Fontan procedure was provided in DICOM format by Our Lady’s Children’s Hospital, Crumlin, Dublin (OLCHC) with some patient data and image parameters given in Table 1. The access to this medical data was approved by the OLCHC ethical board.

**Table 1:** Patient characteristics and imaging details

Age (years)	Sex	BSA ^a^ (m^**2**^)	Pixel size (mm^**2**^)	Slice Thickness (mm)
3.7	Male	0.53	0.78	0.5

^a^Body Surface Area

**Fig. 1:**
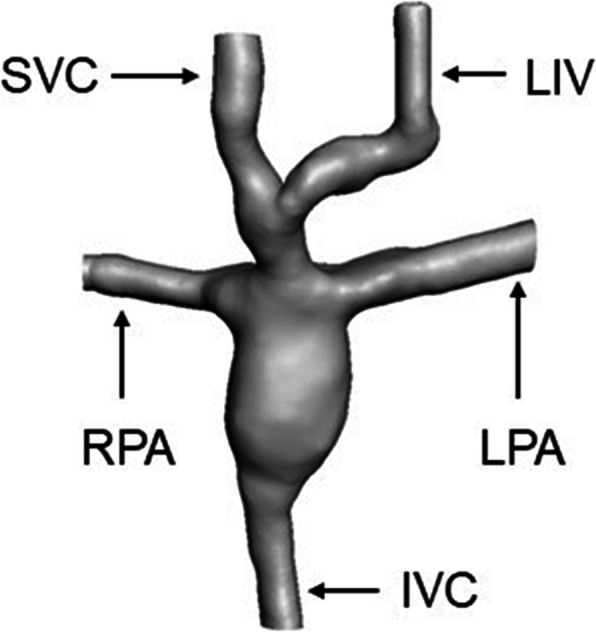
3D Model of the segmented model

The 3D model was generated by segmentation procedures within the open source image reconstruction software 3D slicer [22]. The region of interest included the SVC, IVC, left pulmonary artery (LPA) and right pulmonary artery (RPA). A 3D surface mesh, enclosing the region of interest was generated based on the segmentation mask. After the manual editing process, all voids were filled and smoothed (Fig. 1), and saved in stereolithography (STL) file format.

Due to the proximity of the left innominate vein (LIV) in relation to the SVC, this vessel was included in the final segmented model.

Table 2 shows the inlet and outlet diameters of each vessel obtained by fiducial markers within the 3D Slicer software.

**Table 2:** Inlet (SVC & IVC) and Outlet (LPA & RPA) Diameters

Vessel	Diameter (mm)
Superior vena cava	11.20
Inferior vena cava	12.06
Left pulmonary artery	12.60
Right pulmonary artery	10.30

### In-vitro model fabrication

**Fig. 2:**
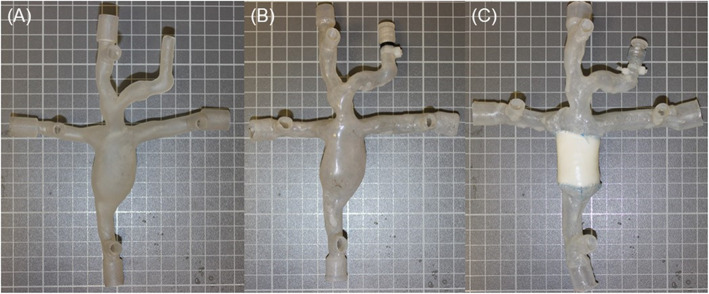
(**a**) Rigid model (**b**) Compliant model (**c**) Compliant model with sutured extracardiac Gore-Tex conduit

Three in-vitro models were fabricated by means of 3D printing and the lost wax methodology similar to arterial models replicated previously in our lab [23, 24]. Inlet and outlet sections were extended a distance of 30 mm to allow for flow connections, which provided a smooth transition with no interference on the flow field. Along with this, pressure ports and tapered connections were included to 1) measure the pressure within the model without inhibiting the flow 2) allow for the internal diameters (ID) of the inlet/outlets to match the inserted tubing, thus creating a seamless connection. One rigid model was fabricated in a Form 2 stereolithography apparatus (SLA) printer (FormLabs, Somerville, MA) using clear resin with a layer thickness of 25 μm. The material consisted of a photo-curable acrylate and epoxy liquid resin. The printed model was further processed by removing support material and cured in a UV chamber (Fig. 2 a). A silicone mixture of Elastosil 4600 part A & B mixed with 10% silicone fluid by weight was used to replicate the arterial wall. This resulted in a Young’s Modulus of 0.25 ± 0.02 MPa replicating the reported stiffness of univentricular anatomy [25]. A thin-walled flexible model was fabricated, by the lost wax methodology. The outer mould was fabricated by SLA with the resulting inner core fabricated by a water soluble polyvinyl alcohol (PVA) by means of fused deposition modelling (FDM). A nominal wall thickness of 1 mm was coated to each model to replicate the in-vivo characteristics [25], as shown in Fig. 2 (b). An extracardiac Gore-Tex conduit (Gore Medical, AZ, USA) was sutured onto another flexible model by (LN) using a 5–0 prolene suture (Ethicon, NJ USA) using a standard over and over surgical knot (Fig. 2 c).

### Physiological waveform replication

**Fig. 3:**
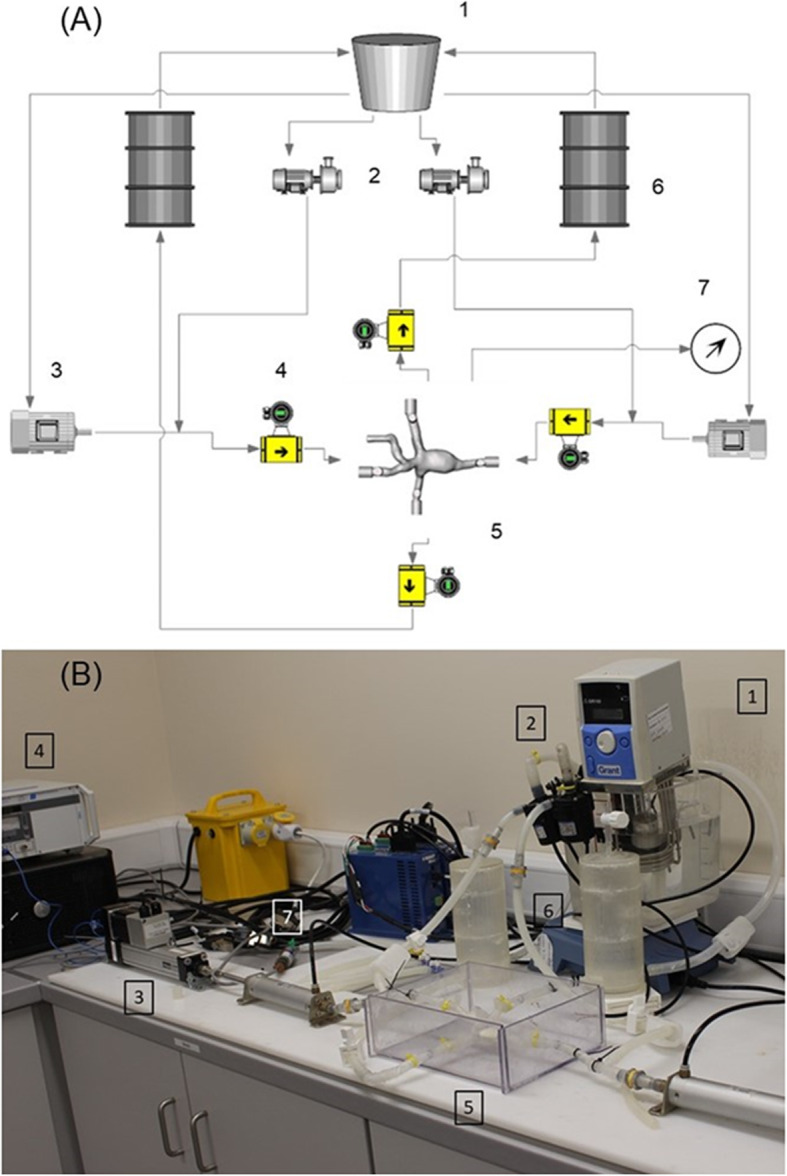
(1) Heated reservoir, (2) Centrifugal pumps, (3) Linear actuators, (4) Flow meters, (5) TCPC Model, (6) Compliance chambers, (7) Pressure transducer

An in-vitro flow rig (IFR) was configured to replicate the flow waveforms (Fig. 3 a & b). Magnetic resonance flow waveform measurements reported in the literature for venae cavae patients with completed Fontans [26] were digitized and replicated using two linear actuators attached to two piston pumps. A data acquisition card (NI USB6001, National Instruments, Berkshire, UK) was used to acquire the input data from the flowrate and pressure sensors with a sampling rate of 5 kHz. A LabVIEW (NI LabVIEW 2018, National Instruments, Berkshire, UK) program was developed to acquire and monitor the pressures and flowrates. The pressure was measured by a pressure transducer manifold (National Instruments NI9205 TX, USA) located at the SVC, IVC, LPA and RPA. The inlet/outlet flowrates were monitored by a clamp on ultrasound flowmeter (Transonics, UK). The selected tubing and blood mimicking fluid were Tygon R-3606 and a glycerine water mix (40:60 ratio by weight). This fluid had a viscosity of 4 ± 0.5 mPa.s as obtained by a cone and plate viscometer (Brookfield, AMETEK, MA USA) which is within the viscosity range of blood [27]. All models where tested in a polycarbonate housing. All measuring instruments where calibrated before each experimental run. No flowrate was prescribed along the LIV due to a lack of flowrate waveform data in literature.

### Physiological flow replication

**Fig. 4:**
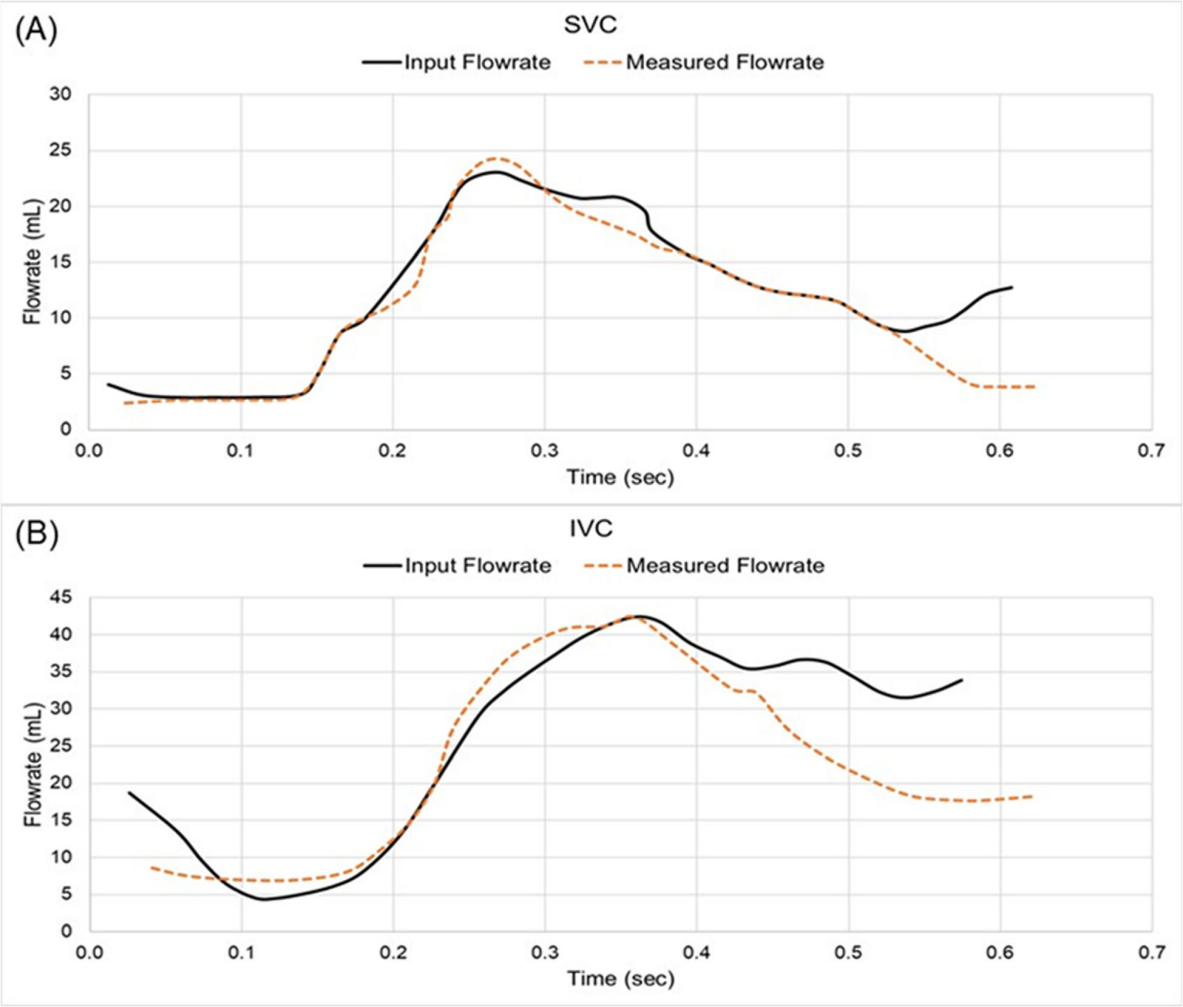
The inputted and measured flow rates for the (A) SVC (B) IVC

The physiological flowrates were replicated (Fig. 4) using a brushless stepper motor (BM75 Nema, Aerotech, Southampton, UK) parallel mounted onto a linear actuator (ET32 Parker Electro thrust cylinder, Parker, OH) and connected to hydraulic cylinders (SMC, Dublin, Ireland) to simulate the pulsatile flow rate through the vasculature as reported by Markl et al., (2011). Centrifugal pumps (Iwaki RD-12, Iwaki America, Holliston, MA) were connected in parallel providing a steady flow feed.

The steady flowrates were defined based off the time averaged flowrates from Markl et al., (2011) with a steady flowrate of 0.65 L/min and 1.65 L/min for the SVC and IVC respectively. Two patient pulsatile flow waveforms were superimposed on the steady flow lines, to replicate the SVC and the IVC flowrates. The flowrates where split by means of turn valve restrictors in the ratio of 60:40 to the LPA and 50:50 as previously investigated [27, 28]. 3D printed windkessels maintained the pressures within physiological limits.

**Fig. 5:**
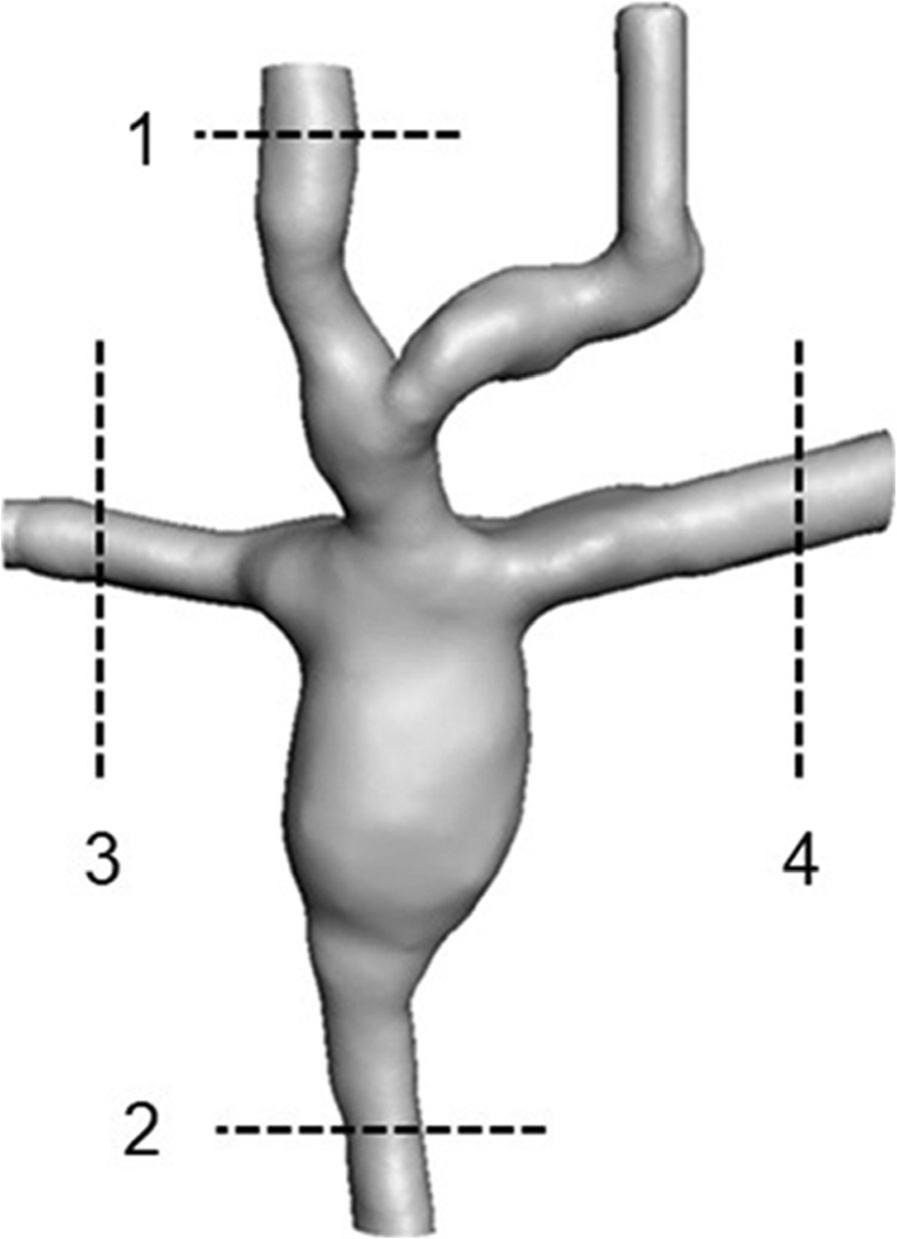
TRANSDUCER LOCATIONS FOR ULTRASOUND TESTING (1) SVC (2) IVC (3) RPA (4) LPA

The water/glycerol solution was seeded with hollow glass beads of 10 μm diameter to mimic red blood cells and increase the colour Doppler (CD) velocity signals. Ultrasound data was collected using a 128-element linear array transducer (12L5V, 5–12 MHz) on a Logiq e (GE Healthcare IL, USA). The transducer was secured perpendicularly to the plane of flow directly above the model. The transducer was placed at four regions of interest, as illustrated in Fig. 5.

### Arterial compliance

Compliance (C) as defined by Tree et al., (2017), is given by:

1 $$ \mathrm{C}=\frac{\Delta  \mathrm{V}}{\Delta  \mathrm{P}} $$

Where ∆V and ∆P are the elemental volume and pressure respectively.

A static pressure test setup was used for the purpose of assessing the thin walled models’ compliance. The suprarenal end of the vascular models was connected with tubing to the pump, while the other end was closed with caps. A T-junction was inserted at the inlet of the vascular model to allow connectivity with the pressure transducer. The volume of fluid from the syringe was measured along with the pressure.

The compliance was measured for each model at the SVC and IVC inlets along with the RPA and LPA outlets. The wall compliance values were 1.34 ± 0.29 mL/mmHg and 1.35 ± 0.43 mL/mmHg for the compliant and Gore-Tex models respectively which is within the anatomical range of 1.36 ± 0.78 mL/mmHg as reported in the literature [25].

### Energy loss

To quantify the fluid dynamic efficiency, the fluid-energy dissipation between the inlet and outlets for all thin walled models were found. The total fluid energy entering the model must equal the fluid energy leaving the model and any incurred losses, in which these energy losses are given by

2 $$ {\mathrm{E}}_{\mathrm{loss}}=\sum {\mathrm{E}}_{\mathrm{in}}-\sum {\mathrm{E}}_{\mathrm{out}} $$

Where the total rate of fluid energy is given by

3 $$ \mathrm{E}=\mathrm{Q}\left\{\mathrm{P}+\frac{1}{2}{\mathrm{pv}}^2\right\} $$

Equation 3 represents both static and kinetic energy contributions, where Q is the flow rate, P is the static pressure, v is the average velocity, and ρ is the fluid density. Combining Eqs. 2 and 3, we arrive at an equation that represents the total energy loss occurring across the vessels for this investigation [28, 29].

4 $$ {\mathrm{E}}_{\mathrm{loss}}=\left\{{\mathrm{Q}}_{\mathrm{svc}}\left\{{\mathrm{P}}_{\mathrm{svc}}+\frac{1}{2}{\uprho \mathrm{v}}_{\mathrm{svc}}^2\right\}+{\mathrm{Q}}_{\mathrm{IVC}}\left\{{\mathrm{P}}_{\mathrm{ivc}}+\frac{1}{2}{\uprho \mathrm{v}}_{\mathrm{ivc}}^2\right\}\right\}-\left\{{\mathrm{Q}}_{\mathrm{LPA}}\left\{{\mathrm{P}}_{\mathrm{LPA}}+\frac{1}{2}{\uprho \mathrm{v}}_{\mathrm{LPA}}^2\right\}+{\mathrm{Q}}_{\mathrm{RPA}}\left\{{\mathrm{P}}_{\mathrm{RPA}}+\frac{1}{2}{\uprho \mathrm{v}}_{\mathrm{RPA}}^2\right\}\right\} $$

## Results

### Pressure waveforms

**Fig. 6:**
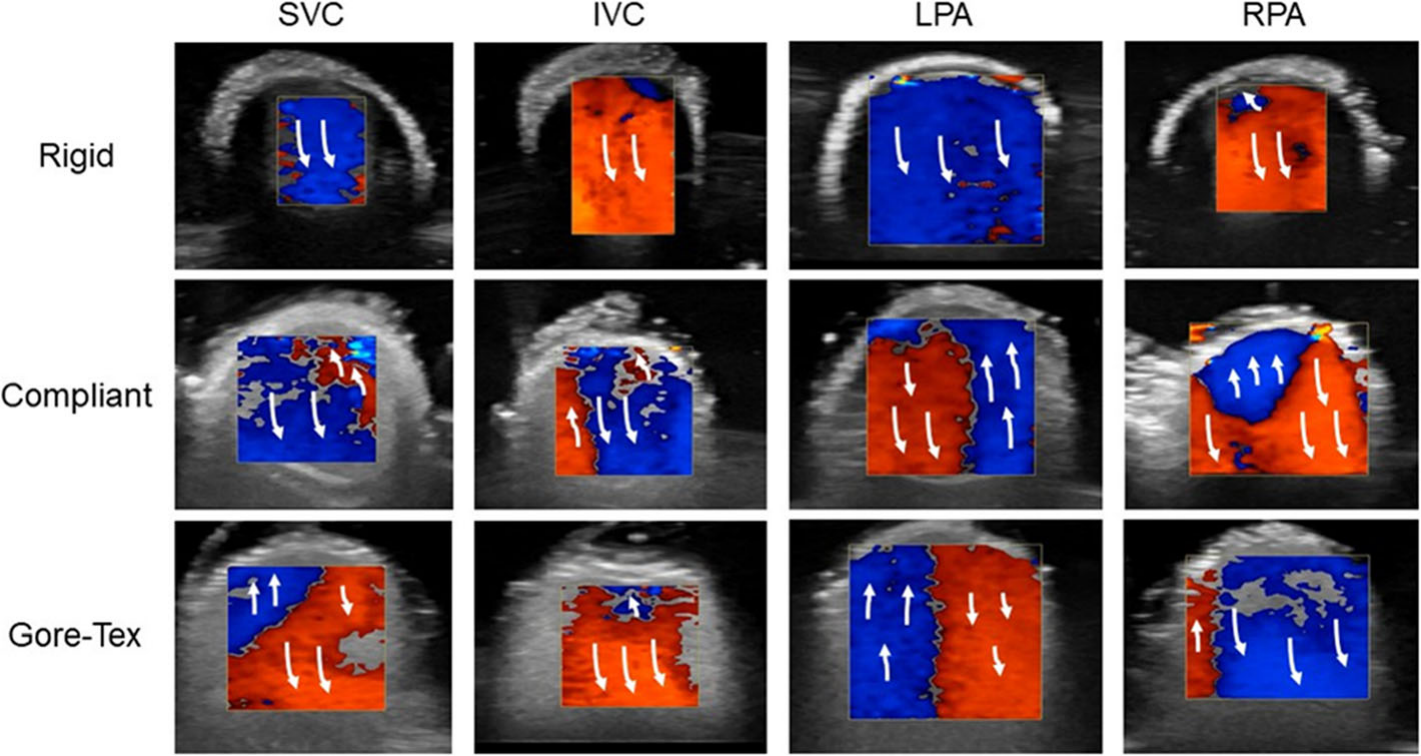
Pressure waveforms for the rigid, complaint and Gore-Tex conduit models for a 60:40 flow split to the LPA

The pressure waveforms for the various models are given in Fig. 6 showing a lower pressure distribution for the compliant walls and Gore-Tex model when compared with the rigid wall model. The compliant wall model had a lower pressure in all regions when compared to the rigid model of 20.27% ± 0.57, 19.08% ± 0.71, 17.49% ± 0.61 and 19% ± 1.2 for the SVC, IVC, LPA and RPA respectively. The same was observed for the Gore-Tex model with lower pressures of 15.98% ± 0.64, 16.80% ± 0.70, 14.52% ± 1.4, and 17.04% ± 2.8 when compared to the rigid model along the SVC, IVC, LPA and RPA respectively. The compliant wall model dampened the pressure waveforms in all regions.

### Energy loss

The mean flowrates and the energy losses for all tests are shown in Tables 3, 4 and 5. In all cases the rigid and compliant models had the lowest and highest energy losses respectively. Table 3 outlines the flowrate and energy losses under a 60:40 pulsatile flow split condition. There were energy loss increases of 26.43 and 18.15% in the compliant and Gore-Tex model compared to the rigid walled model respectively. The Gore-Tex model had a lower energy loss of 8.38%, when compared to the compliant model.

**Table 3:** Flow rate and energy loss for the rigid, compliant and Gore-Tex walled models using a pulsatile flow rate with a 60:40 flow split to the LPA. Q is the flow rate

	Rigid	Compliant	Gore-Tex
Q_SVC_ (L/min)	0.65 ± 0.01	0.65 ± 0.01	0.65 ± 0.01
Q_IVC_ (L/min)	1.65 ± 0.01	1.65 ± 0.01	1.65 ± 0.01
Q_RPA_ (L/min)	1.38 ± 0.01	1.38 ± 0.01	1.39 ± 0.01
Q_LPA_ (L/min)	0.88 ± 0.01	0.9 ± 0.01	0.9 ± 0.01
Energy Loss (mW)	**5.91**	**7.71**	**7.09**

**Table 4:** Flow rate and energy loss for the rigid, compliant and Gore-Tex walled models using a pulsatile flow rate with a 50:50 flow split. Q is the flow rate

	Rigid	Compliant	Gore-Tex
Q_SVC_ (L/min)	0.65 ± 0.01	0.65 ± 0.01	0.65 ± 0.01
Q_IVC_ (L/min)	1.65 ± 0.01	1.65 ± 0.01	1.65 ± 0.01
Q_RPA_ (L/min)	1.16 ± 0.01	1.15 ± 0.01	1.15 ± 0.01
Q_LPA_ (L/min)	1.14 ± 0.01	1.15 ± 0.01	1.14 ± 0.01
Energy Loss (mW)	**8.18**	**10.31**	**9.54**

**Table 5:** Flow rate and energy loss for the rigid, compliant and Gore-Tex walled models using a steady flow rate with a 60:40 flow split to the RPA. Q is the flow rate

	Rigid	Compliant	Gore-Tex
Q_SVC_ (L/min)	0.65 ± 0.01	0.65 ± 0.01	0.65 ± 0.01
Q_IVC_ (L/min)	1.65 ± 0.01	1.65 ± 0.01	1.65 ± 0.01
Q_RPA_ (L/min)	1.39 ± 0.01	1.39 ± 0.01	1.38 ± 0.01
Q_LPA_ (L/min)	0.9 ± 0.01	0.9 ± 0.01	0.88 ± 0.01
Energy Loss (mW)	**4.89**	**5.93**	**5.18**

Table 4 shows the 50:50 flow split to the pulmonary arteries using the patient specific pulsatile waveform. All scenarios had greater levels of energy losses compared to the 60:40 flow split. An increase of 23.04 and 15.35% in energy loss was found in the complaint and Gore-Tex models when compared to the rigid model, with the compliant model having 7.76% increase in energy loss when compared to the Gore-Tex.

Table 5 shows the resulting energy losses when a steady flow was used for both inlets with a 60:40 flow split to the RPA. As can be seen in Table 5, a percentage increase in the compliant model of 19.22% and Gore-Tex of 5.76% when compared to the rigid walled model. An increase of 13.50% occurred when comparing the Gore-Tex energy loss to the complaint model.

### Flow visualisation

**Fig. 7:**
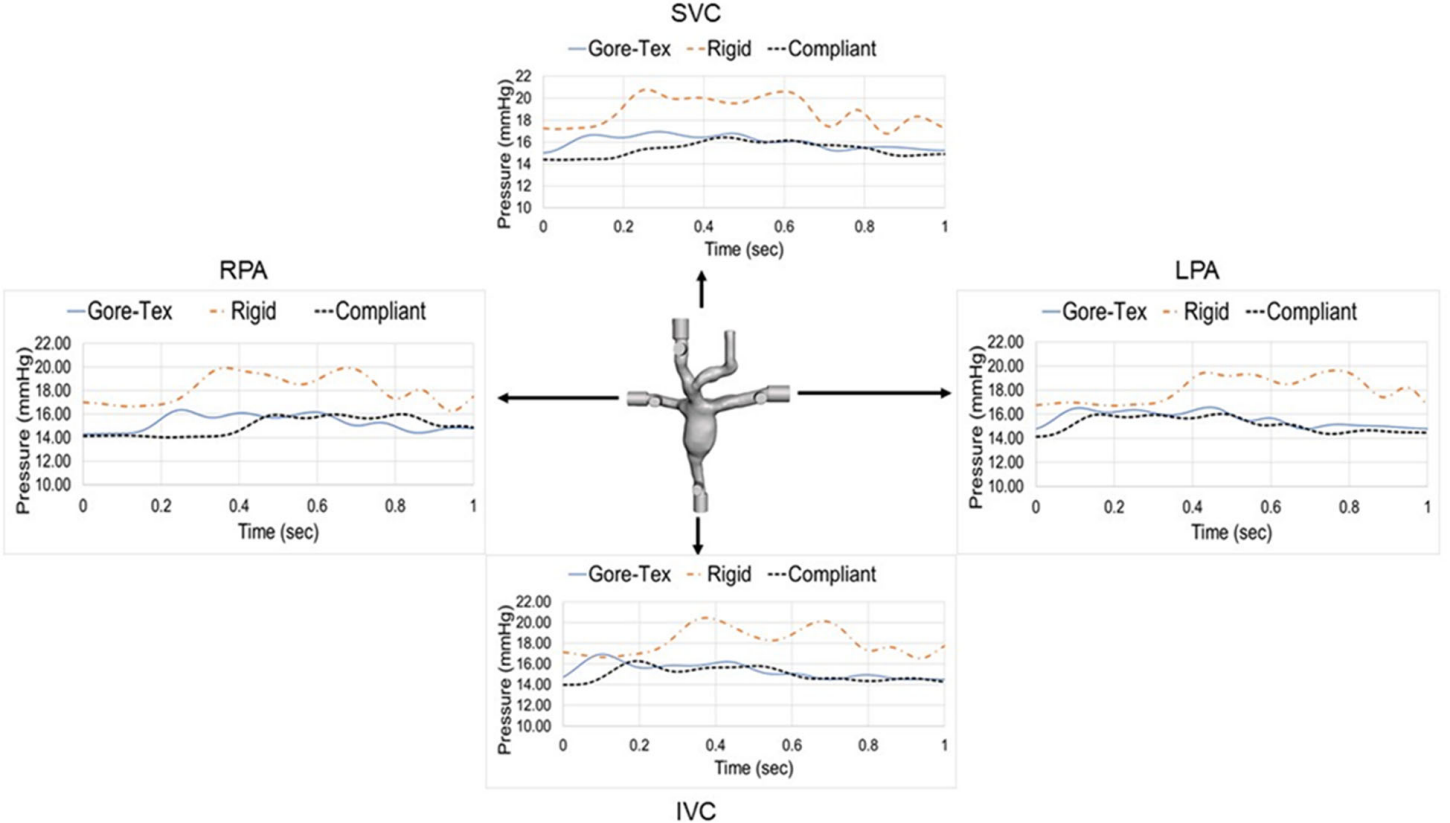
Ultrasound results of various TCPC models, the top row shows the rigid models, the middle row contains the thin walled flexible model and below the Gore-Tex model

The Colour Doppler images of the various models are shown in Fig. 7 for the 60:40 flow split to the LPA. Red and blue indicate the directionality of the flow towards and away from the probe respectively. Areas of high levels of mixing (turbulent) were represented by the mosaic of colours. Each image presented in Fig. 7 was captured during the systolic phase. The coloured Doppler illustrated that a greater level of turbulence was observed in each vessel for the compliant and Gore-Tex models when compared to the rigid model, this was observed along the SVC, IVC, LPA, and RPA as shown in Fig. 7. The compliant and Gore-Tex walled models had similar flow patterns along the SVC and LPA with the complaint model having slightly more turbulence/mixing at the IVC and RPA regions.

## Discussion

This study we believe is the first of its kind in assessing the haemodynamic performance of a commercially available extracardiac conduit sutured within a thin walled flexible TCPC patient specific phantom model. The conduit set-up was compared with a rigid and flexible wall models, which was a useful comparison in assessing performance. To date, there are very few studies simulating compliant walls for TCPC simulations.

This investigation has shown that when comparing rigid walls and compliant walls in this particular anatomy a significant change occurs in the flow hemodynamics. This aspect has been noted as a limitation for previous studies assuming no significant difference in the flow hemodynamics with the incorporation of rigid walls [15–17]. Long et al., (2012) completed a fluid structure interaction (FSI) study to identify the impact of compliance on the Fontan procedure and found minimal difference between rigid and deformable walls in terms of energy losses of less than 2% but noted material properties of the anatomy were not available at the time and incorporated a mean pressure rather than pulsatile. Our findings found a significant difference in energy losses with rigid walls underestimating the energy loss by 21–30%. Our study incorporated reported vascular properties [25] and produced pulsatile pressure waveforms using a replicated patient specific waveform, which may explain these differences.

Our findings showed that the compliant and Gore-Tex walled models had increased levels of energy loss compared to the rigid model ranging from 5 to 27%. When comparing the energy losses of the compliant model to the Gore-Tex there was an increase of 7 to 14%. The energy losses were underestimated by 18–60% when comparing steady flow with pulsatile flow and differing outlet percentage flow splits. These results are in agreement with the experimental findings of Chitra et al., (2009) for comparing steady versus pulsatile flow in an idealised TCPC model.

This study compared the fluid dynamic efficiency of rigid, complaint and a sutured Gore-Tex model and offers further insights into possible optimization of flows by the integration of compliant walls. Results from these experiments suggest that the inclusion of compliance into patient specific in-vitro models provides the best overall replication of the TCPC for future geometry and graft studies.

Pulmonary flow splits are anywhere near 55%:45% (RPA:LPA) in healthy humans and are derived from the mass ratio of the right and left lungs (ie, more blood flows normally through the bigger right lung) [30]. Early in-vitro studies with different simplified models have shown that the least energy loss is observed with 45 to 55% blood flow to the RPA, with increasing energy loss when flow splits are highly skewed toward one of the lungs [28, 29] . However, these values only apply for these specific models and conditions because models with other geometric properties show other optimal flow splits, ranging from 30 to 70% RPA flow [31, 32]. For example, de Zelicourt et al. (2006) showed the least energy loss for a 70% RPA flow split because this patient specific model had a small LPA. Increased flow through this LPA resulted in increased energy loss. Additionally, Whitehead et al. (2007) reported increased energy loss for most patients when flow toward the LPA increased because in most patients the LPA is smaller than the RPA. However, in some patients, the effect of altering pulmonary flow split only had a minor effect on energy loss, and in others increasing LPA flow split resulted in decreased energy loss. In these latter patients, relative RPA hypoplasia was present. Restoring PA diameter will restore a more balanced pulmonary flow split [33]. An increase in energy losses occurred for all model scenarios by changing the flow split to the pulmonary arteries from 60:40 to the LPA to an even split 50:50 for both pulmonary arteries, with observed increases of 29–32%.

The ultrasound analysis allowed us to compare the flow fields of the rigid complaint and Gore-Tex walled models. It was found that when comparing the flow fields of the rigid model against both compliant and surgical scenarios that less turbulence and mixing was observed throughout all vessels. When comparing the complaint and Gore-Tex models minimal differences occurred in terms of turbulence and mixing, with the Gore-Tex model performing minimally better due to less mixing within the IVC and RPA.

While the in-vitro experimental approach allowed us to reproduce the major physiologic parameters of the TCPC circulation, there are limitations worth noting. Respiratory-induced alterations in pulmonary flow were not modelled. Although this is not expected to affect relative performance between procedures, it is possible that respiratory-induced pulsatility may affect absolute hemodynamic efficiency for each model and will be investigated in future experiments.

## Conclusion

The compliant walled model replicating a native tissue graft in-vivo scenario and Gore-Tex surgical model gave increased energy losses of 19–26% and 5–18% respectively when compared with the baseline rigid model. When the compliant model was compared to the Gore-Tex surgical scenario, there was an increase of 7–13% in energy losses demonstrating the overall benefit of the conduit, in decreasing energy losses. The ultrasound analysis showed that the rigid walled model experienced less turbulence and mixing compared to the compliant walled models, with minimal difference in the compliant and Gore-Tex models.

## Data Availability

All supporting data is readily available upon request.
